# Discovery and Preclinical Development of SM15685, a Novel Highly Selective Oral DYRK1A/B Inhibitor as a Potential Therapeutic for Alzheimer’s Disease

**DOI:** 10.1002/alz.087795

**Published:** 2025-01-09

**Authors:** Chi‐Ching Mak, Gopi Mittapalli, Chelsea Nora, Emily Creger, Lewis Turner, Brian Eastman, Brian Hofilena, Ramkrishna Vakiti, David Benardo, Graeme Taylor, John S Hill, Carine Bossard

**Affiliations:** ^1^ Biosplice Therapeutics, Inc., San Diego, CA USA

## Abstract

**Background:**

DYRK1A overexpression, common in neurodegenerative diseases like Alzheimer’s (AD), contributes to neurofibrillary tangles via Tau protein hyperphosphorylation and amyloid plaque formation, key AD hallmarks. Therefore, DYRK1A has been regarded as a novel target for neurodegenerative diseases. However, developing DYRK1A selective inhibitors has been a difficult challenge due to the highly conserved ATP‐binding site of protein kinases, particularly among the CMGC family. Here we describe SM15685, a novel highly selective and potent oral DYRK1A/B inhibitor, which reduces Tau hyperphosphorylation both in vitro and in vivo.

**Method:**

Compound selectivity and potency were evaluated in kinase panels and cellular target engagement assays. Tau phosphorylation (pTau) was measured in cell‐based assays. Pharmacokinetics (PK) were evaluated in rodents and pharmacodynamics evaluated in 3‐month‐old JNPL3 female mice (P301L human Tau overexpression) treated orally with SM15685 or Vehicle for 7 days (7 mice/group). pTau at Thr212 was biochemically quantified in subcortical brain and spinal cord fractions.

**Result:**

SM15685 was developed by utilizing structure‐based drug design and via iterative medicinal chemistry optimization cycles to achieve high potency, selectivity against the closely related CLKs and GSK3β, and good oral PK profiles characterized by brain‐penetration. SM15685 selectively and potently inhibited DYRK1A kinase activity (IC_50_ = 1 nM) with >90% inhibition at 1μM and only inhibited <2% of the full kinome. Selectivity and potency for DYRK1A and DYRK1B were confirmed in cellular target engagement assays (IC_50_ = 18 and 55 nM, respectively). SM15685 reduced pTau at the Threonine 212 site (IC_50_ = 14 nM). In pharmacokinetic studies, SM15685 demonstrated high oral bioavailability (%F >70) and good brain‐penetration in rodents (Kpuu >0.6). Compared to vehicle, JNPL3 mice treated with SM15685 at 45mg/kg showed significant reduction of pTau in both subcortical and spinal cord fractions in a pharmacodynamic model (p<0.05, T‐test).

**Conclusion:**

SM15685, a highly selective and potent, oral, brain‐penetrant, DYRK1A/B inhibitor with the ability to significantly reduce Tau phosphorylation, has potential as a treatment for chronic tauopathies such as AD. These studies support further evaluation of SM15685 as a therapeutic option for AD and other diseases characterized by Tau hyperphosphorylation.

